# Controlled synthesis of various Fe_2_O_3_ morphologies as energy storage materials

**DOI:** 10.1038/s41598-021-84755-z

**Published:** 2021-03-04

**Authors:** Bui Thi Hang, Trinh Tuan Anh

**Affiliations:** International Training Institute for Materials Science, Hanoi University of Science and Technology, No. 1, Dai Co Viet Road, Hanoi, Viet Nam

**Keywords:** Energy science and technology, Materials science

## Abstract

Air pollution from vehicle emissions is a major problem in developing countries. Consequently, the use of iron-based rechargeable batteries, which is an effective method of reducing air pollution, have been extensively studied for electric vehicles. The structures and morphologies of iron particles significantly affect the cycle performance of iron-based rechargeable batteries. The synthesis parameters for these iron materials also remarkably influence their structures, shapes, sizes, and electrochemical properties. In this study, we fabricated α-Fe_2_O_3_ materials with various shapes and sizes via a facile hydrothermal route and investigated the effects of raw materials on their structures, morphologies, and properties. The structural characteristics of the synthesized iron oxides were studied via X-ray diffraction using scanning electron microscopy. Results indicate that changing the concentration of raw materials modified the structure and morphology of the synthesized α-Fe_2_O_3_ particles, that is, the desired shape and size of α-Fe_2_O_3_ can be controlled. The effects of the structure and morphology of α-Fe_2_O_3_ particles on their electrochemical characteristics were investigated. The results show that the morphology and shape of the iron oxide particles remarkably affected the redox reaction rate and discharge capacity of the Fe_2_O_3_/C composite electrodes. Among the synthesized α-Fe_2_O_3_ materials, the cubic-shaped α-Fe_2_O_3_ exhibited the highest discharge capacity. This material is a potential candidate for application in iron-based aqueous batteries. Our results may facilitate not only the controlled synthesis of α-Fe_2_O_3_ nanoparticles for potential technical applications but also the production of electrode materials with high capacity and good cycle performance for iron-based rechargeable batteries.

## Introduction

Metal–air batteries have attracted the attention of researchers because they have a high theoretical energy, are environment-friendly, and cost lower than lithium-ion batteries^1–5^. Among the metal–air batteries, iron–air batteries are the most valued because iron has high theoretical specific energy and low cost and is abundant on earth and often non-toxic^6–10^. However, the actual energy of iron–air batteries has remained low due the limitations of iron electrodes, such as large overpotential, the passivation caused by the iron hydroxide formed during discharge, and low coulombic efficiency. In addition, hydrogen evolves simultaneously with reduction reaction of iron lowers the charging efficiency results in loss of water from the electrolyte^11–13^.

In aqueous alkaline solution, two plateaus can be observed in the discharge process. The first plateau at =  − 0*.*975 V versus Hg*/*HgO corresponds to the oxidation of iron to Fe(OH)_2_. The second plateau at − 0*.*758 V versus Hg*/*HgO or − 0*.*658 V versus Hg*/*HgO corresponds to the further oxidation of Fe(OH)_2_ to Fe(III). Iron (III) can be formed as Fe_3_O_4_, FeOOH, and/or Fe_2_O_3_^11,14,15^:

1 $${\text{Fe}} + {\text{2OH}}^{ - } \leftrightarrow {\text{Fe}}\left( {{\text{OH}}} \right)_{{2}} + {\text{2e}}^{ - } ,E_{{\text{o}}} = - 0.{\text{975 V versus}}\;{\text{Hg/HgO}},$$

2 $${\text{3Fe}}\left( {{\text{OH}}} \right)_{{2}} + {\text{2OH}}^{ - } \leftrightarrow {\text{Fe}}_{{3}} {\text{O}}_{{4}} + {\text{4H}}_{{2}} {\text{O}} + {\text{2e}}^{ - } ,E_{{\text{o}}} = - 0.{\text{758 V versus}}\;{\text{Hg/HgO}},$$

and/or

3 $${\text{Fe}}\left( {{\text{OH}}} \right)_{{2}} + {\text{OH}}^{ - } \leftrightarrow {\text{FeOOH}} + {\text{H}}_{{2}} {\text{O}} + {\text{e}}^{ - } ,E_{{\text{o}}} = \, - 0.{\text{658 V versus}}\;{\text{Hg/HgO}}.$$

The low electrical conductivity of the iron oxides formed during the discharge processes can result in the low coulombic efficiency of iron electrodes.

The hydrogen evolution reaction is expressed as follows:

4 $${\text{2H}}_{{2}} {\text{O}} + {\text{2e}}^{ - } \leftrightarrow {\text{H}}_{{2}} + {\text{2OH}}^{ - } ,E_{{\text{o}}} = - 0.{926}\;{\text{V versus Hg/HgO}}.$$

The standard reduction potential of the hydrogen evolution in alkaline electrolyte (Eq. 4) is slightly more positive than that of the iron electrode reaction (Eq. 1). Hydrogen evolution is possible even at open circuit on an iron electrode in alkaline solution. Consequently, hydrogen evolution (Eq. 4) occurs during charging (Eq. 1) in addition to the reduction in the iron electrode. Simultaneous hydrogen evolution can result in low faradaic efficiency during charging.

Previous studies^16–20^ reported the following reactions of Fe_2_O_3_ electrodes in alkaline solution at the first charge:

5 $${\text{Fe}}_{{2}} {\text{O}}_{{3}} + {\text{3H}}_{{2}} {\text{O}} + {\text{2e}}^{ - } \to {\text{2Fe}}\left( {{\text{OH}}} \right)_{{2}} + {\text{2OH}}^{ - } ,$$

and

6 $${\text{Fe}}\left( {{\text{OH}}} \right)_{{2}} + {\text{2e}}^{ - } \to {\text{Fe}} + {\text{2OH}}^{ - } .$$

The composition and structure of iron electrodes have been modified to address their limitations. Furthermore, some additives for electrodes and/or electrolyte solutions are used to increase the redox reaction rate and reduce the rate of hydrogen evolution, resulting in the improved capacity and efficiency of iron electrodes^21–29^. However, the limitations of iron electrodes have not been overcome completely, so more investigations are needed. Previous studies have shown that the structure and morphology of iron particles strongly influence their electrochemical properties in alkaline solutions^24–32^. In the present study, we synthesized α-Fe_2_O_3_ materials with various structures and morphologies using a hydrothermal route to find the most suitable material for iron-based aqueous batteries. The desired shape and size of α-Fe_2_O_3_ particles were controlled by changing the concentration of raw materials and the hydrothermal parameters. Furthermore, we applied additives to the electrolyte to further improve the capacity and cycle performance of iron electrodes.

## Results and discussion

**Figure 1:**
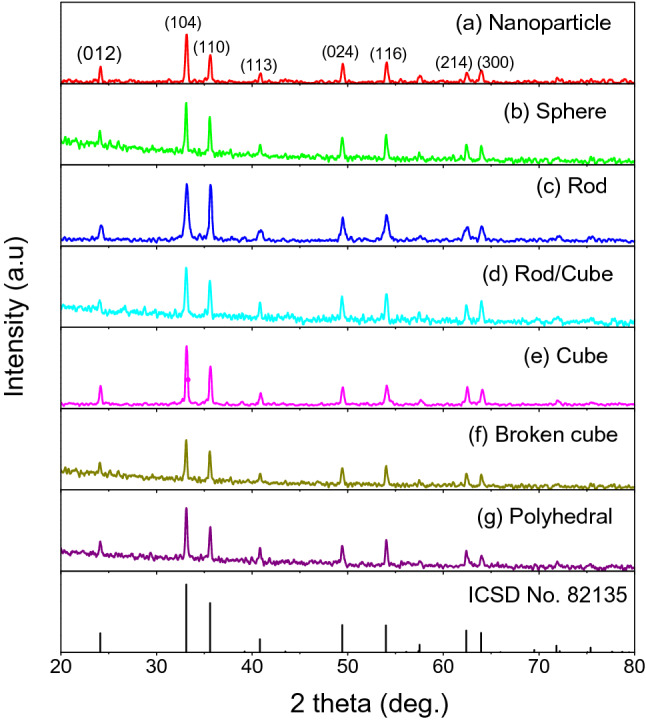
XRD patterns of α-Fe_2_O_3_ synthesized via hydrothermal route at various concentration ratios of FeCl_3_ and cetyltrimethylammonium bromide (CTAB) ($${C}_{{FeCl}_{3}}$$/$${C}_{CTAB})$$: (**a**) $${C}_{{FeCl}_{3}}$$/$${C}_{CTAB}=0.02/0.0$$, (**b**) $$0.02/0.01$$, (**c**) $$0.05/0.01$$, (**d**) $$0.05/0.02$$, (**e**) $$0.05/0.04$$, (**f**) $$0.07/0.04$$, (**g**) $$0.03/0.04$$.

Figure 1 shows the X-ray patterns of the as-prepared materials. The patterns of all the samples exhibit some typical diffraction peaks at 2 theta values of 24.17°, 33.19°, 35.66°, 40.9°, 49.51°, 54.13°, 62.49°, and 64.05°, which respectively correspond to the (012), (104) (110), (113), (024), (116), (214), and (300) planes. All the peaks can be easily indexed to hematite α-Fe_2_O_3_ and are consistent with the reported values in ICSD No. 82135. No other peaks for impurities can be observed in the XRD patterns. This result confirms that the synthesized products are pure α-Fe_2_O_3_.

From the Scherer equation, the mean size of hematite α-Fe_2_O_3_ crystallites is calculated, and the results are shown in Table 1. The concentration ratio of iron chloride and the CTAB surfactant (${C}_{{FeCl}_{3}}$/${C}_{CTAB}$) affects the average size of hematite crystallites. Without the CTAB (${C}_{{FeCl}_{3}}$/${C}_{CTAB}=0.02/0.0$), the mean size of hematite crystallites is approximately 29 nm (Fig. 1a). Using the CTAB with ${C}_{CTAB}=0.01 M$, if ${C}_{{FeCl}_{3}}=0.02 M$ (${C}_{{FeCl}_{3}}$/${C}_{CTAB}$=0.02/0.01) (Fig. 1b), the mean size of hematite crystallites increases. However, if enhancing the concentration of FeCl_3_ to 0.05 M, the mean size of hematite crystallites decreases (Fig. 1c). The mean size of hematite crystallites increases when ${C}_{{FeCl}_{3}}=0.05 M$ is maintained and the CTAB amount is increased to 0.02 M or 0.04 M, that is, ${C}_{{FeCl}_{3}}$/${C}_{CTAB}=0.05/0.02$ (Fig. 1d) or ${C}_{{FeCl}_{3}}$/${C}_{CTAB}=0.05/0.04$ (Fig. 1e). Similarly, the mean size of hematite crystallites increases when ${C}_{CTAB}=0.04 M$ is maintained and the concentration of iron chloride is increased or decreased, that is, ${C}_{{FeCl}_{3}}$/${C}_{CTAB}=0.07/0.04$ (Fig. 1f) or ${C}_{{FeCl}_{3}}$/${C}_{CTAB}=0.03/0.04$ (Fig. 1g). Thus, the concentrations of iron chloride and the CTAB surfactant affect the mean size of hematite crystallites. The synthesized *α*-Fe_2_O_3_ hematite crystallites are polycrystalline materials whose particles contain several crystals.

**Table 1:** Concentration ratios of precursors, mean size of the hematite crystallites synthesized via hydrothermal route.

No.	Sample names	Concentration ratios of precursors ($${C}_{{FeCl}_{3}}$$/$${C}_{CTAB})$$	Mean size of the hematite crystallites
1	Nanoparticle	${C}_{{FeCl}_{3}}$/${C}_{CTAB}=0.02/0.0$	29.04
2	Sphere	${C}_{{FeCl}_{3}}$/${C}_{CTAB}=0.02/0.01$	36.36
3	Rod	${C}_{{FeCl}_{3}}$/${C}_{CTAB}=0.05/0.01$	20.52
4	Rod/Cube	${C}_{{FeCl}_{3}}$/${C}_{CTAB}=0.05/0.02$	30.68
5	Cube	${C}_{{FeCl}_{3}}$/${C}_{CTAB}=0.05/0.04$	33.08
6	Broken cube	${C}_{{FeCl}_{3}}$/${C}_{CTAB}=0.07/0.04$	36.41
7	Polyhehral	${C}_{{FeCl}_{3}}$/${C}_{CTAB}=0.03/0.04$	35.36

**Figure 2:**
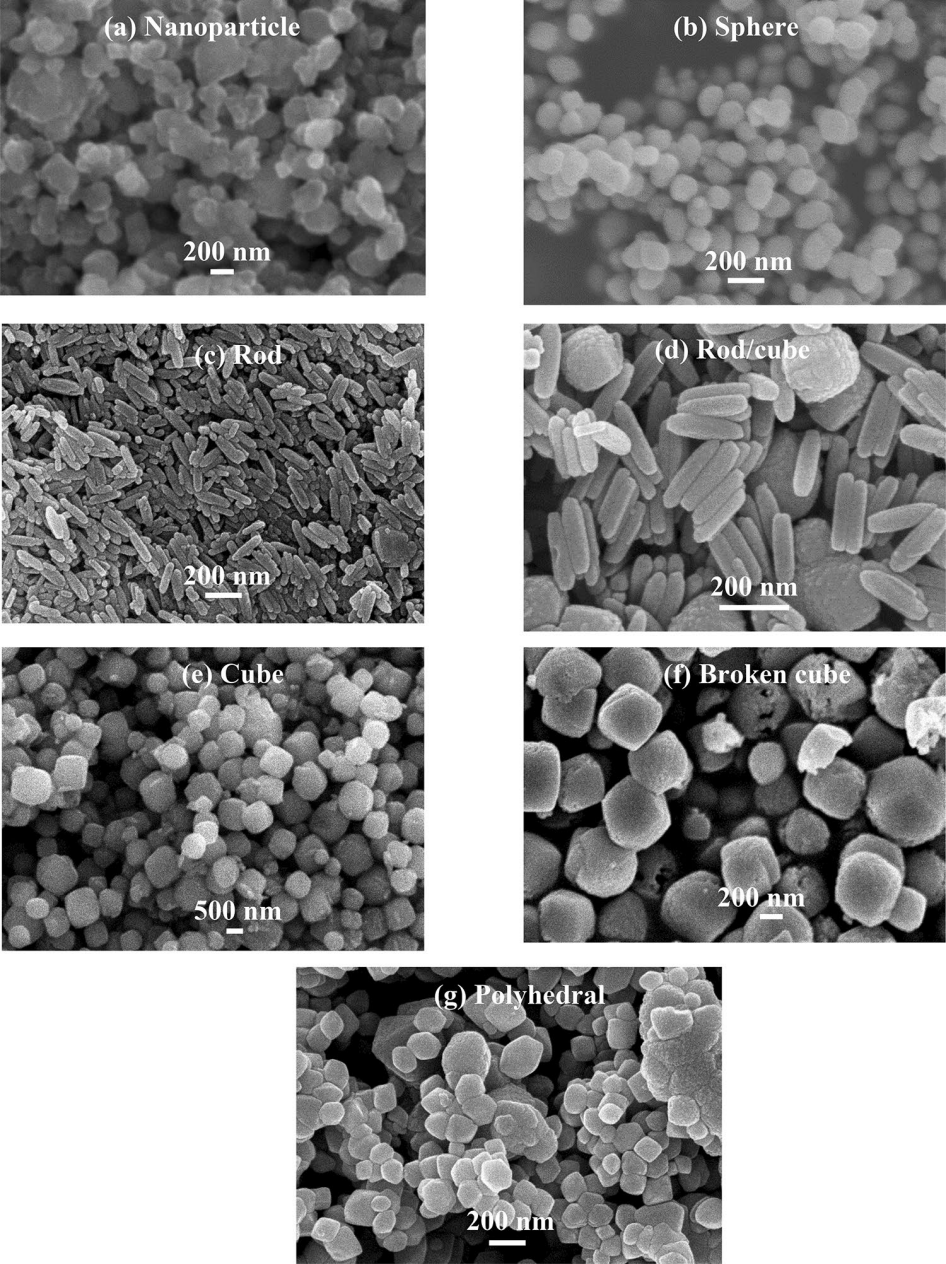
SEM images of the α-Fe_2_O_3_ materials synthesized at various concentration ratios of FeCl_3_ and CTAB: (**a**) ${C}_{{FeCl}_{3}}$/${C}_{CTAB}=0.02/0.0$(nanoparticle), (**b**) $0.02/0.01$(sphere), (**c**) $0.05/0.01$ (rod), (**d**) $0.05/0.02$ (rod/cube), (**e**) $0.05/0.04$ (cube), (**f**) $0.07/0.04$ (broken cube), (**g**) $0.03/0.04$ (polyhedral).

The SEM images of the α-Fe_2_O_3_ materials synthesized at various concentration ratios of FeCl_3_ and CTAB are shown in Fig. 2. All the samples were prepared under the same hydrothermal conditions, namely, at 120 °C for 14 h and a pH value of 10 in a typical hydrothermal process, but the concentration ratio of precursors FeCl_3_ and CTAB (${C}_{{FeCl}_{3}}$/${C}_{CTAB}$) was changed to obtain Fe_2_O_3_ materials with different morphologies, shapes, and sizes.

In this study, CTAB acts as a cationic surfactant during synthesis. Thus, the CTAB amount affects the morphological characteristics of the synthesized iron oxides. Furthermore, the concentration of iron chloride also affects the morphology of the obtained iron oxides. By adjusting the concentration ratio of ${C}_{{FeCl}_{3}}$/${C}_{CTAB}$ in the reaction solution, different morphologies of *α*-Fe_2_O_3_ nanoparticles can be obtained. Pu et al.^33^ showed that the *α*-Fe_2_O_3_ formation in the hydrothermal process may proceed through two steps. First, Fe^3+^ is hydrolyzed in aqueous solution to form the *β*-FeOOH precursor. Second, the resulting *β*-FeOOH undergoes topotactic transformation to *α*-Fe_2_O_3_ via the dissolution/reprecipitation mechanism. They demonstrated that the morphologies of the *β*-FeOOH precursors change continuously from nanosphere, nanorod, to nanoraft with the increase of the FeCl_3_ concentration under the confinement of surfactants.

Figure 2a presents the sample prepared with ${C}_{{FeCl}_{3}}$= 0.02 M without CTAB (${C}_{{FeCl}_{3}}$/${C}_{CTAB}=0.02/0.0$) is nanostructured particles, free shape, non-uniform with diameters from a few hundred nanometers to one micrometer. When ${C}_{CTAB}=0.01 M$ and ${C}_{{FeCl}_{3}}=0.02 M$ (${C}_{{FeCl}_{3}}$/${C}_{CTAB}$=0.02/0.01) (Fig. 2b), sphere-shaped iron oxides are formed with diameters less than 200 nm. When ${C}_{CTAB}=0.01 M$ and the iron chloride amount is increased to 0.05 M, that is, ${C}_{{FeCl}_{3}}$/${C}_{CTAB}=0.05/0.01$, the product comprises rod-like nanoparticles (Fig. 2c). The diameters of the *α*-Fe_2_O_3_ nanorods are approximately 50 nm, and their lengths range from 100 to 200 nm. When ${C}_{{FeCl}_{3}}=0.05 M$ and the CTAB concentration is increased to ${C}_{CTAB}=0.02 M$, nanorods aggregate and thus form some nanocubes in the product (Fig. 2d). Notably, when ${C}_{CTAB}=0.04 M$, perfect cubic-shaped structures are observed in the sample (Fig. 2e), but their dimensions are not uniform, with edges of a few hundred nanometers to one micrometer. Thus, when ${C}_{{FeCl}_{3}}$ is low at ${C}_{CTAB}=0.01 M$, the product mainly consists of zero-dimensional nanoparticles, such as nanospheres (Fig. 2b). According to Pu et al.^33^, the precursors in this case might be sphere-like particles of several nanometers. The shapes of the synthesized products turn to rods (Fig. 2c) and cubes (Fig. 2e) with the gradual increase of the FeCl_3_ concentration. The cubes are attributed to be transformed from raft-like precursors or aggregation of nanorods. The product containing both rods and cubes (Fig. 2d) is an intermediate step of turning from rods to cubes, suggesting that the precursor morphologies change from sphere to rod to cube with the increase of ${C}_{{FeCl}_{3}}$. This transformation is like that observed by Pu et al.^33^. When ${C}_{CTAB}=0.04 M$ and the iron chloride amount is increased to 0.07 M, that is, ${C}_{{FeCl}_{3}}$/${C}_{CTAB}=0.07/0.04$, the product still consists of nanocubes, and some of them are broken (Fig. 2f). When the iron chloride concentration is decreased to ${C}_{{FeCl}_{3}}=0.03 M$ while ${C}_{CTAB}=0.04 M$ (${C}_{{FeCl}_{3}}$/${C}_{CTAB}=0.03/0.04$), the perfect cubic-shaped structures of Fe_2_O_3_ are destroyed, the sample contains non-uniform polyhedral particles, and the particle sizes are less than one micrometer.

Consequently, when the FeCl_3_ concentration is low, sphere-like *β*-FeOOH precursors of several nanometers and capped completely by CTAB are formed and transformed into sphere-shaped *α*-Fe_2_O_3_ during precursor dissolution/reprecipitation. By increasing the amount of FeCl_3_ to moderate, *α*-Fe_2_O_3_ nanorods form via the transformation of rod-like *β*-FeOOH precursors. When the FeCl_3_ concentration is high, the precursors turn to larger raft-like particles, which aggregate and transform to cubic-shaped *α*-Fe_2_O_3_particles. These results indicate that the shapes of the *β*-FeOOH precursors have a significant effect on the morphologies of the synthesized *α*-Fe_2_O_3_, and CTAB plays an important role in mediating the hydrolysis of FeCl_3_. CTAB-the cationic surfactant-capping agents can confine the growth of iron(III) oxides in the nanometer regime. The concentration ratios of raw materials FeCl_3_ and CTAB ($${C}_{{FeCl}_{3}}$$/$${C}_{CTAB})$$, the sample names, and the preparation conditions are listed in Table 2.

**Table 2:** Preparation conditions and samples synthesized via hydrothermal route.

No.	Sample names	Concentration ratios of precursors ($${C}_{{FeCl}_{3}}$$/$${C}_{CTAB})$$	Hydrothermal treatment
1	Nanoparticle	${C}_{{FeCl}_{3}}$/${C}_{CTAB}=0.02/0.0$	120 °C—14 h, pH 10
2	Sphere	${C}_{{FeCl}_{3}}$/${C}_{CTAB}=0.02/0.01$	120 °C—14 h, pH 10
3	Rod	${C}_{{FeCl}_{3}}$/${C}_{CTAB}=0.05/0.01$	120 °C—14 h, pH 10
4	Rod/Cube	${C}_{{FeCl}_{3}}$/${C}_{CTAB}=0.05/0.02$	120 °C—14 h, pH 10
5	Cube	${C}_{{FeCl}_{3}}$/${C}_{CTAB}=0.05/0.04$	120 °C—14 h, pH 10
6	Broken cube	${C}_{{FeCl}_{3}}$/${C}_{CTAB}=0.07/0.04$	120 °C—14 h, pH 10
7	Polyhehral	${C}_{{FeCl}_{3}}$/${C}_{CTAB}=0.03/0.04$	120 °C—14 h, pH 10

In this case, the hydrothermal parameters, namely, pH value, time, and temperature reaction, were kept constant with the changing concentrations of the raw materials, namely, ${C}_{CTAB}$ and ${C}_{{FeCl}_{3}}$. In the presence of CTAB, *α*-Fe_2_O_3_ nanoparticles with different shapes were obtained when ${C}_{{FeCl}_{3}}$ was changed. When ${C}_{{FeCl}_{3}}=0.02$, *α*-Fe_2_O_3_ nanospheres formed. The shapes of the synthesized *α*-Fe_2_O_3_ turned to rods (Fig. 2c) and then cubes (Fig. 2e) with the gradual increase of the FeCl_3_ concentration. When CTAB was not used, the particles grew randomly to form nanoparticles with different shapes and sizes (Fig. 2a). At ${C}_{{FeCl}_{3}}=0.02$, *α*-Fe_2_O_3_ nanospheres appeared when ${C}_{CTAB}=0.01$. When the CTAB concentration was increased to 0.04 and ${C}_{{FeCl}_{3}}=0.05$, the morphologies of *α*-Fe_2_O_3_ changed from spheres to rods and then to cubes. When ${C}_{CTAB}=0.04$, the morphology of the *α*-Fe_2_O_3_ particles changed to imperfect cubes, that is, broken (Fig. 2f) or polyhedral (Fig. 2g) cubes, with the further increase or decrease of the FeCl_3_ concentration. This result confirms that CTAB can confine the growth of synthesized products in the nanometer regime, while the FeCl_3_ concentration can change the shape of the *α*-Fe_2_O_3_ nanoparticles. These results are consistent with those in the literature^33^. Therefore, the morphological characteristics of α-Fe_2_O_3_ are controlled by changing the concentration of raw materials. The synthesized α-Fe_2_O_3_ nanoparticles with various morphologies can be applied in various technologies. The α-Fe_2_O_3_ particles with perfect cubic-shaped structures are expected to improve the electrochemical properties of the iron-based battery anode.

**Figure 3:**
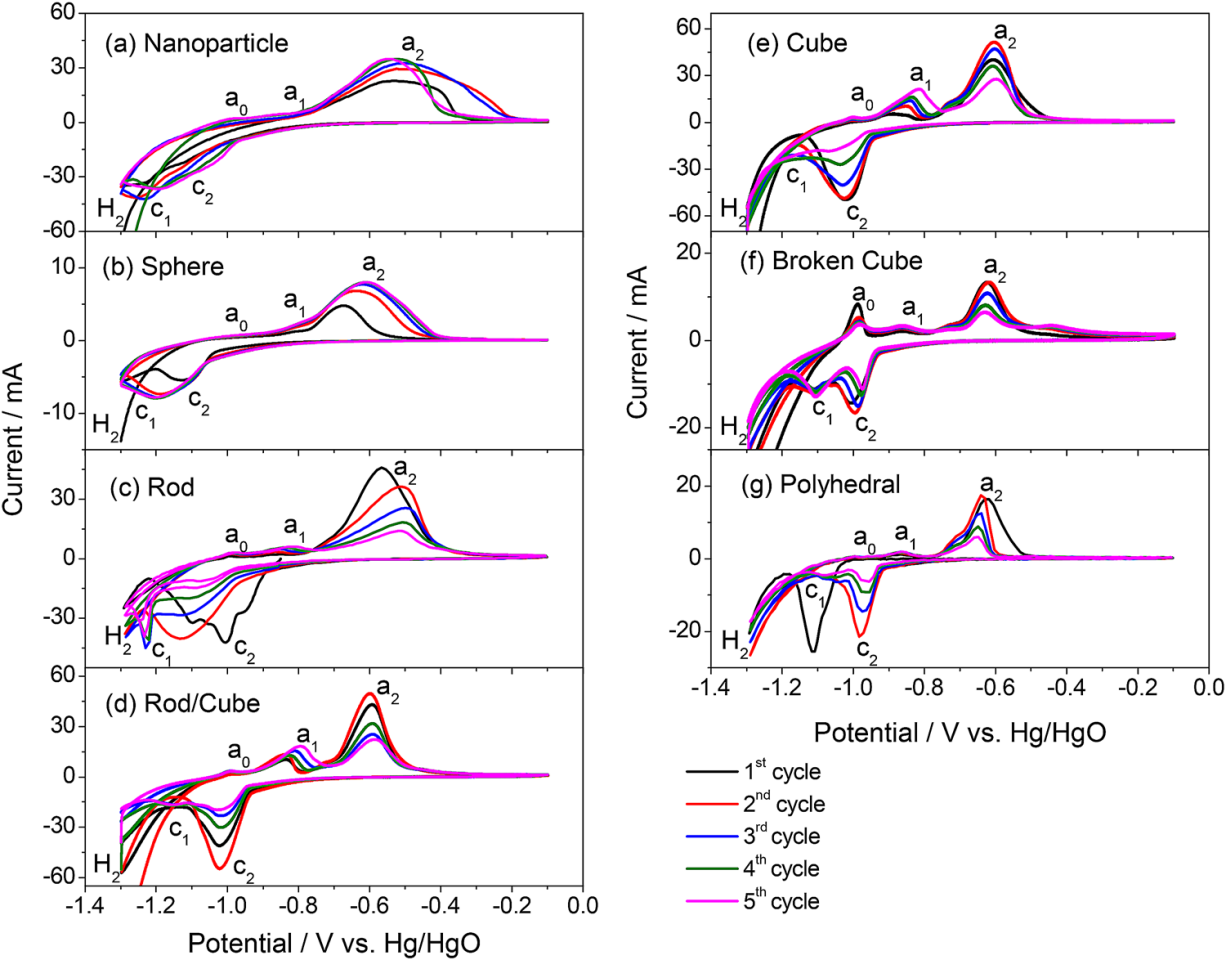
CVs of Fe_2_O_3_/AB electrodes with various morphologies of Fe_2_O_3_ synthesized via hydrothermal route in KOH solution: (**a**) nanoparticle, (**b**) sphere, (**c**) rod, (**d**) rod/cube, (**e**) cube, (**f**) broken cube, and (**g**) polyhedral.

The cyclic voltammetry (CV) measurements of the Fe_2_O_3_/AB electrodes in base electrolyte were taken to investigate the electrochemical properties of the α-Fe_2_O_3_ materials fabricated via a hydrothermal route, and the results are presented in Fig. 3. The CV profiles of the samples are similar, except that of the Fe_2_O_3_/AB electrodes using the Fe_2_O_3_ nanoparticle obtained at ${C}_{{FeCl}_{3}}$/${C}_{CTAB}=0.02/0.0$ (Fig. 3a). The CV results of the samples depict two pairs of redox peaks, namely, Fe/Fe(II) (a_1_/c_1_) and Fe(II)/Fe(III) (a_2_/c_2_), observed at approximately − 0.8 V (a_1_)/ − 1.1 V (c_1_) and − 0.6 V (a_2_)/ − 1.0 V (c_2_). Anodic peaks *a*_1_ and *a*_2_ can be attributed to the oxidation of Fe to Fe(II) and Fe(II) to Fe(III), while cathodic peaks *c*_2_ and *c*_1_ correspond to the reduction of Fe(III)/Fe(II) and Fe(II)/Fe, respectively. Thus, *a*_1_ and *c*_1_ correspond to the Fe/Fe(II) redox couple (Eq. 1), while *a*_2_ and *c*_2_ correspond to the Fe(II)/Fe(III) redox couple (Eqs. 2 or 3). Furthermore, we also observed a small oxidation peak (*a*_0_) around − 1.0 V and hydrogen evolution around − 1.2 V. According to Cerny et al.^15^, the first oxidation peak (*a*_0_) corresponds to iron oxidation to form [Fe(OH)]_ads_ before Fe(OH)_2_ is produced. Oxidation peak a_2_ is relatively larger and broader than a_1_, especially for the Fe_2_O_3_ nanoparticles (Fig. 3a), and the redox current under the a_2_/c_2_ couple is higher than that under the a_1_/c_1_ couple, suggesting that anodic peak *a*_2_ covers the oxidation of the Fe/Fe(II) and Fe(II)/Fe(III) couples. Remarkably, the reduction peaks (c_1_) of the Fe_2_O_3_/AB electrodes with Fe_2_O_3_ nanoparticles (Fig. 3a) and sphere (Fig. 3b) are completely covered by hydrogen evolution peaks, which may result in low charge efficiency.

Further cycling, the oxidation peaks shifted toward a more positive potential, while the reduction peaks moved toward a more negative potential with the Fe_2_O_3_ nanoparticles (Fig. 3a), sphere (Fig. 3b), and rod (Fig. 3c) samples; this shift increased the overpotentials, leading to the disappearance of redox peaks. In contrast, the overpotentials gradually decreased with the Fe_2_O_3_ rods/cubes (Fig. 3d), cubes (Fig. 3e), broken cubes (Fig. 3f), and polyhedral particles (Fig. 3g). The increase in overpotential may cause negative effects on the capacity and cycling efficiency of Fe_2_O_3_/AB electrodes. In addition, the redox currents of the electrodes with Fe_2_O_3_ nanoparticles (Fig. 3a) and sphere (Fig. 3b) increased upon cycling and decreased with the remaining Fe_2_O_3_ samples (Fig. 3c–f).

The CV profiles of the electrodes with Fe_2_O_3_ rode/cube, cube, broken cube, and polyhedral samples are similar (Fig. 3d–g); for instance, the redox current increased and then decreased, and their reduction peaks (c_1_) are separated from the hydrogen evolution peaks. This behavior is quite different from that of the Fe_2_O_3_ nanoparticle and sphere samples (Fig. 3a,b), that is, the redox current gradually increased, and their reduction peaks (c_1_) disappeared. In contrast, the Fe_2_O_3_ rod (Fig. 3c) seems to be the intermediate state of the two types of CV curves. These CV results are consistent with the observation of the SEM results as evidenced by the Fe_2_O_3_ rode/cube, cube, broken cube, and polyhedral samples (Fig. 2d–g), which have similar morphologies, while the Fe_2_O_3_ nanoparticle and sphere samples (Fig. 2a,b) have similar morphologies, and the rod (Fig. 2c) is in intermediate shape. However, the cubic-shaped structures of the Fe_2_O_3_ in the polyhedral sample (Fig. 2g) are destroyed, exhibiting random polyhedral particle shapes. Therefore, they provide smaller redox peaks (Fig. 3g) than other cubic-shaped Fe_2_O_3_ samples (Fig. 3d–f). These findings suggest that the morphological characteristics of the Fe_2_O_3_ synthesized via a hydrothermal route significantly affect the electrochemical properties of Fe_2_O_3_/AB electrodes.

A comparison of the CV results of all samples Fe_2_O_3_ nanoparticle, sphere, rod, rod/cube, cube, broken cube and polyhedral shows that cubic-shaped Fe_2_O_3_ provides better cyclability than the other samples as clearly evidenced by the well-defined redox peaks. This phenomenon is acceptable because the morphological characteristics of iron oxide affect its redox behaviors. At similar cubic shapes, the Fe_2_O_3_ particles in the electrode are close-packed, and the contact between cubic-shaped Fe_2_O_3_ and AB should be tighter than that between other-shaped Fe_2_O_3_ and AB. Thus, the internal resistance of the cubic-shaped Fe_2_O_3_/AB electrode should be smaller than that of the other-shaped Fe_2_O_3_/AB electrode. Consequently, the reaction rate of the cubic-shaped Fe_2_O_3_ particles was higher than that of other-shaped Fe_2_O_3_ particles. The SEM images of the synthesized Fe_2_O_3_ materials show that Fe_2_O_3_ nanoparticles (Fig. 2a), spheres (Fig. 2b), and rods (Fig. 2c) have a smaller size than cubic-shaped Fe_2_O_3_ (Fig. 2d–g) and thus need more binders to prepare Fe_2_O_3_/AB electrodes. However, all the electrodes were prepared at the same ratio of Fe_2_O_3_:AB:PTFE = 45:45:10 wt%. The number of binders was insufficient to tightly bind to iron oxide and the AB particles in the electrodes when a smaller Fe_2_O_3_ size was used, resulting in the high internal resistance of the Fe_2_O_3_/AB electrode. Consequently, Fe_2_O_3_/AB electrodes with Fe_2_O_3_ nanoparticles, spheres, and rods exhibit a low redox reaction rate, leading to increased overpotentials and unobservable peaks, whereas the cubic-shaped Fe_2_O_3_ provides better cyclability and observable oxidation peaks (c_1_).

Thus, the Fe_2_O_3_ materials synthesized at different hydrothermal conditions exhibited different morphologies and consequently provided different electrochemical characterizations. In other words, hydrothermal parameters affected the particle sizes and shapes of the Fe_2_O_3_ materials and strongly influenced their electrochemical properties. Therefore, by changing the preparation conditions, we can control the desired morphologies, sizes, and shapes of the Fe_2_O_3_ materials to provide the best electrochemical characterizations.

The CV results show that the shape, size, and morphology of the Fe_2_O_3_ particles strongly affected the electrochemical properties of Fe_2_O_3_/AB electrodes, and the cubic-shaped Fe_2_O_3_, including rods/cubes, cubes, and broken cubes, exhibited better cyclability than the Fe_2_O_3_ nanoparticles, spheres, and rods.

**Figure 4:**
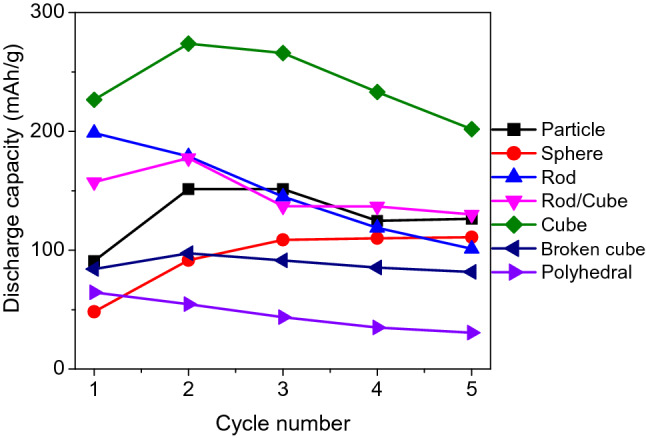
Discharge capacities of Fe_2_O_3_/AB electrodes in KOH solution.

To find the most suitable Fe_2_O_3_ among the synthesized materials, the discharge capacities of the Fe_2_O_3_/AB electrodes with all the synthesized Fe_2_O_3_ were calculated from the CV profiles, and the results are shown in Fig. 4. Among the synthesized materials, the Fe_2_O_3_ cube provided the largest discharge capacities. This result is acceptable from the perspective that among the cubic-shaped Fe_2_O_3_, the Fe_2_O_3_ cubes possessed a perfect cubic shape, whereas the Fe_2_O_3_ rods/cubes contained rods and the broken cubes fragments of cubes. This result confirms that Fe_2_O_3_ cube is the most suitable material. However, the discharge capacity of the Fe_2_O_3_/AB electrode with an Fe_2_O_3_ cube gradually decreased with repeated cycling. The electrolyte additive was used to overcome this problem.

**Figure 5:**
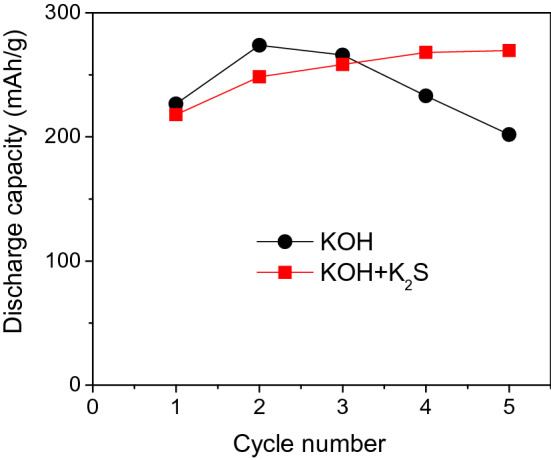
Discharge capacities of Fe_2_O_3_/AB electrodes with Fe_2_O_3_ cube in KOH and KOH + K_2_S solutions.

The effects of the K_2_S additive on the electrochemical properties of the Fe_2_O_3_/AB composite electrode were investigated with the Fe_2_O_3_ cube, showing the highest capacity (Fig. 4), and the result is shown in Fig. 5. The Fe_2_O_3_ cube sample in the additive electrolyte has larger discharge capacity than that in the base electrolyte. The positive effects of the S^2−^ additive have been reported in previous studies as follows. Ion sulfide is incorporated into the oxide lattice and interacts with Fe(I), Fe(II), or Fe(III) in the oxide film to promote the dissolution of iron^24,28^, thus preventing the rapid passivation of iron electrodes during discharge, and increasing the hydrogen evolution overpotential and extending the discharge curve^13,24–29^, thereby improving the cyclability and the discharge rates.

**Figure 6:**
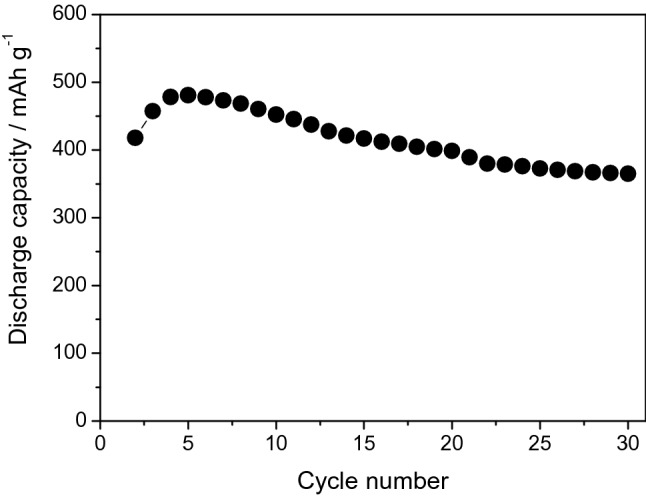
Discharge capacities of Fe_2_O_3_/AB electrode with cubic-shaped α-Fe_2_O_3_ in KOH + K_2_S solution.

The charge–discharge measurement was taken in KOH aqueous solution containing K_2_S additive to evaluate the applicability of α-Fe_2_O_3_ cube materials synthesized via a hydrothermal route in rechargeable batteries. Figure 6 presents the discharge capacities as a function of the number of cycles for the Fe_2_O_3_/AB electrode using α-Fe_2_O_3_ cube in the additive electrolyte. The discharge capacities increased from 418 mAh g^−1^ in the first cycle to 457, 478, and 481 mAh g^−1^ in the second, third, and fourth cycles, respectively, and then gradually decreased to 478 mAh g^−1^ in the fifth cycle. The discharge capacity of the Fe_2_O_3_/AB electrode was approximately 365 mAhg^−1^ at the 30th cycle. Thus, the discharge capacities gradually decreased with the number of cycles. This capacity decline is ascribed to the passivation of the Fe(OH)_2_ layer formed during the discharge process even in presence of K_2_S additive in the electrolyte. Yang et al.^28^ investigated sintered iron electrodes and found that the electrode surface is packed closely, and the pores are also filled with the discharge products. In the inner part of the electrode, the discharge products appeared less tightly packed, and a considerable number of pores remained available. They also demonstrated that with small pores, a larger surface of the active material is available for the electrode reaction during discharge. In our case, the Fe_2_O_3_/AB composite electrode was prepared by pressing a mixture of the Fe_2_O_3_ particles, AB, and PTFE binder, resulting in high porosity and small pores. It also has a large active material surface for better electrode reaction during discharge. However, capacity decline still occurs and is attributed to the passivation of the Fe(OH)_2_ layer formed during the discharge process. At the first cycle, the discharged product (Fe(OH)_2_) is formed on the outer parts of the electrode, and the interior parts of the electrode become inaccessible to the electrolyte. Consequently, the iron oxide particles in the interior of the electrode remain under-utilized. Given the small pores of the electrode, in the next cycles, the electrolyte diffuses into the inner parts of the electrode, and redox reactions occur, thereby increasing the capacity. However, the redox reactions of the inner portions of the porous electrodes are poor. With further cycling, the pores are filled with the Fe(OH)_2_ product, blocking the electrolyte access. Consequently, the capacity gradually decreases.

**Figure 7:**
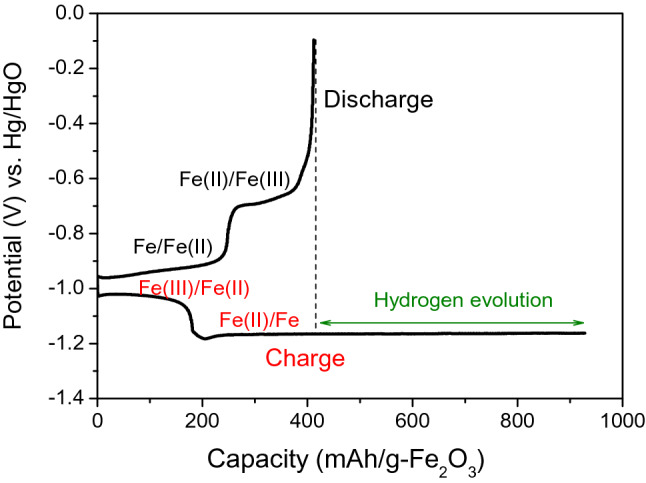
Charge–discharge curves of Fe_2_O_3_/AB electrode with cubic-shaped α-Fe_2_O_3_ in KOH + K_2_S.

As mentioned above, when K_2_S was added to the electrolyte, S^2-^ additives interacted with Fe(I), Fe(II), and Fe(III) in the oxide film to promote the dissolution of iron, increasing the hydrogen evolution overpotential and prolonging the discharge curve, thereby improving the cyclability and discharge rate of iron electrodes. From the charge–discharge curves, we can estimate the hydrogen evolution produced during the charge and the contribution of the Fe/Fe(II) and Fe(II)/Fe(III) redox couples to the discharge capacity of the electrode. Therefore, the charge–discharge measurement of the Fe_2_O_3_/AB electrode additive was taken in the additive electrolyte, and the results are shown in Fig. 7.

The charge capacity is much larger than the discharge capacity. Two plateaus are observed at approximately − 0.95 and − 0.70 V (vs. Hg/HgO) in the discharge curves corresponding to the Fe/Fe(II) (Eq. 1) and Fe(II)/Fe(III) (Eqs. 2 and/or 3) reactions, whereas they occur at approximately − 0.95 and − 1.15 V (vs. Hg/HgO) in the charge curves. During the charging process, the valence of iron changes from Fe(III) to Fe(II) and then Fe(II) to Fe. The length of the first and second plateaus in the discharge curves is nearly the same. In the charge curves, the second plateau is longer than the first plateau, suggesting that the second plateau covered the reduction reaction of Fe(II)/Fe (Eq. 1) and hydrogen evolution (Eq. 4). The difference between the discharge and charge capacities is ascribed to the hydrogen evolution reaction. This result demonstrates that hydrogen evolution was only partly suppressed by the S^2-^ additive. The simultaneous hydrogen evolution with the iron deposition reaction results in a low faradaic efficiency during charging and discharge capacity deterioration. Thus, capacity decline is related to the passivation of the Fe(OH)_2_ formed during the discharge process and the hydrogen evolution reaction during the charge process.

With the further improvement in the capacity retention of Fe_2_O_3_/AB composite electrodes, the synthesized cubic-shaped α-Fe_2_O_3_ material can be a potential candidate for energy storage systems. Furthermore, the α-Fe_2_O_3_ nanoparticles with various morphologies synthesized via a facile hydrothermal route can also be potential materials for other technical applications.

## Conclusion

In the present study, various morphologies of α-Fe_2_O_3_, including nanoparticle, sphere, rod, rod/cube, cube, broken cube, and polyhedral shapes, were successfully synthesized via a facile hydrothermal route in which the morphology is controllable by changing the concentration of raw materials FeCl_3_ and CTAB. CTAB acts as a surfactant and capping agent and FeCl_3_ acts as the iron source during synthesis; thus, their concentrations affect the morphological characteristics of the synthesized iron oxides. CTAB can confine the growth of the synthesized α-Fe_2_O_3_ in the nanometer regime, while the concentration of FeCl_3_ changes the shape of the *α*-Fe_2_O_3_ nanoparticles. By adjusting the concentration ratio of ${C}_{{FeCl}_{3}}$/${C}_{CTAB}$ in the reaction solution, various morphologies of *α*-Fe_2_O_3_ materials could be produced. These α-Fe_2_O_3_ nanoparticles present potential applications in various technological areas.

The investigation of the electrochemical behaviors of the Fe_2_O_3_/AB composite electrodes using synthesized *α*-Fe_2_O_3_ revealed that the morphology, shape, and size of Fe_2_O_3_ particles strongly affect the performance of Fe_2_O_3_/AB electrodes. Among the various synthesized α-Fe_2_O_3_, the cubic shape displayed the highest discharge capacity, but the discharge capacity gradually decreased with prolonged cycling. The K_2_S additive in the electrolyte was used to improve the capacity decline. The positive effect of the K_2_S additive on the Fe_2_O_3_/AB electrodes was confirmed by the good cyclability and large capacity of the Fe_2_O_3_/AB electrodes in the additive electrolyte. Through the optimization of the fabrication conditions and the further improvement in capacity retention, cubic-shaped α-Fe_2_O_3_ synthesized via hydrothermal treatment is a potential material for iron-based battery anodes. The controllable synthesis of α-Fe_2_O_3_ nanoparticles via a facile hydrothermal route is a potential method of producing a large number of nanomaterials for various applications.

## Experimental

### Synthesis of α-Fe_2_O_3_ nanoparticles with various morphologies

We prepared *α*-Fe_2_O_3_ nanoparticles with different sizes and shapes using the method reported in literature^33^ with modifications. The chemicals used in this work, including iron chloride (FeCl_3_.6H_2_O) and cetyltrimethylammonium bromide (CTAB), were of analytical grade. CTAB was used as a surfactant and iron chloride as the iron source. Initially, CTAB aqueous solutions with various concentrations (${C}_{CTAB}$) of 0.01, 0.02, and 0.04 M were prepared by dissolving corresponding CTAB amounts in deionized water, stirring the mixture until the solution became transparent. Iron chloride aqueous solutions with various concentrations (${C}_{{FeCl}_{3}}$) of 0.02, 0.03, 0.05, and 0.07 M were prepared using the same process. Then, 80 ml of iron chloride aqueous solution was dissolved in 80 ml of the above CTAB aqueous solution with various concentration ratios of FeCl_3_/CTAB (${C}_{{FeCl}_{3}}$/${C}_{CTAB}$), namely, 0.02/0, 0.02/0.01, 0.05/0.01, 0.05/0.02, 0.05/0.04, 0.07/0.04, and 0.03/0.04. The resultant mixtures were stirred for 60 min to form homogeneous solutions, and the pH value was adjusted to 10 using NH_3_ before they were transferred into a Teflon-lined stainless-steel autoclave, sealed, and maintained at 120 °C for 14 h in a typical hydrothermal process. The autoclave was then gradually cooled down to room temperature. The red brown precipitates were collected through centrifugation, washed several times with distilled water and ethanol, subsequently dried at 60 °C for 12 h, and finally annealed at 400 °C for 2 h in air to obtain α-Fe_2_O_3_ powder.

### Characterization

The crystal structure of the synthesized powder was identified using an X-ray diffractometer (XRD; Rigaku) with Cu Kα radiation (k = 0.1542 nm) at 40 kV and 150 mA. The diffraction pattern was recorded in the 2-theta range of 20°–80° with a scanning rate of 5°/min. The morphology and shape of the as-synthesized products were observed by scanning electron microscopy (SEM, JEOL JSM-6060LA/VI, Japan) with an accelerating voltage of 25 kV.

### Electrode preparation

The electrode sheet was prepared by mixing 45 wt% synthesized Fe_2_O_3_ powder as an active material, 45 wt% acetylene black carbon (AB, Denki Kagaku Co.) as an additive, and 10 wt% polytetrafluoroethylene (PTFE; Daikin Co.) binder, followed by rolling. The Fe_2_O_3_/AB composite electrodes were punched from the electrode sheets into the pellets of 1-cm diameter. The electrode was then pressed onto the conductive Titanium mesh with a pressure of approximately 150 kg cm^−2^.

### Electrochemical measurements

The electrochemical measurements are described in our previous work^32,34^. In this study, we applied the charge current density of 50 mA cm^−2^ instead of 5 mA cm^−2^ in the cycling performance measurement. The details of the electrochemical measurements are as follows. CV and charge/discharge measurements were taken on a three-electrode glass cell assembly with an Fe_2_O_3_/AB composite as the working electrode, Pt mesh as the counter electrode, and Hg/HgO as the reference electrode. The electrolyte was either a base electrolyte or an additive electrolyte. The base electrolyte was 8 mol dm^−3^ of aqueous KOH solution, and the additive electrolyte was a KOH solution containing 0.01 M K_2_S. The CV measurements were taken at a scan rate of 5 mV s^-1^ and within the range of − 1.3 V to − 0.1 V.

For the cycling performance measurement, the galvanostatic process with a cutoff capacity of 1007 mAh g^−1^ Fe_2_O_3_ was carried out in the charge course. The applied charge current density was 50 mA cm^−2^. In the discharge course, a constant current density of 2.0 mAcm^−2^ was applied to the electrode with a cutoff potential of − 0.1 V. In all the electrochemical measurements, fresh electrodes were used without precycling.
